# Patient-reported outcome after oncoplastic breast surgery compared with conventional breast-conserving surgery in breast cancer

**DOI:** 10.1007/s10549-020-05544-2

**Published:** 2020-01-27

**Authors:** Michael Rose, Henry Svensson, Jürgen Handler, Ute Hoyer, Anita Ringberg, Jonas Manjer

**Affiliations:** 1 Department of Surgery, Section of Plastic Surgery, Hospital of Southwest Jutland, Finsensgade 35, 6700 Esbjerg, Denmark; 2 Department of Clinical Sciences Malmö, Lund University, Malmö, Sweden; 3 Department of Plastic and Reconstructive Surgery, Skåne University Hospital, Malmö, Sweden; 4 Department of Surgery, Section of Breast Surgery, Hospital of South Jutland, Åbenrå, Denmark; 5 Department of Breast Surgery, Ålborg University Hospital, Ålborg, Denmark; 6 Department of Surgery, Skåne University Hospital, Malmö, Sweden

**Keywords:** Oncoplastic breast surgery, Breast conserving surgery, Patient-reported outcome, Breast-Q, Breast cancer

## Abstract

**Introduction:**

Oncoplastic breast surgery (OBS) has developed as an extension of breast-conserving surgery (BCS) in an effort to improve esthetic and functional outcome following surgery for breast cancer. The aim of the present study was to evaluate the possible benefits of OBS, as compared with BCS, with regard to health-related quality of life (HRQoL), using patient-reported outcome measures (PROMs).

**Patients and methods:**

Patients treated with OBS (*n* = 200) and BCS (*n* = 1304) in the period 1 January 2008 to 31 December 2013 were identified in a research database and in the Danish Breast Cancer Cooperative Group (DBCG) registry. Data on patient, tumor, and treatment characteristics were retrieved from the DBCG registry. Patients were sent a survey including the Breast-Q™ BCT postoperative module and a study-specific questionnaire (SSQ) in 2016. A good outcome in the Breast-Q module was defined as above the median. OBS was compared to BCS using a logistic regression analysis, and then adjusted for potential confounders, yielding odds ratios (OR) with 95% confidence intervals.

**Results:**

There was a statistically significant better outcome considering the HRQoL domain “Psychosocial Well-being “ for patients treated with OBS as compared with BCS (OR 2.15: 1.25–3.69). No statistically significant differences were found for the domains “Physical Well-being” (0.83: 0.50–1.39), “Satisfaction with Breast” (0.95: 0.57–1.59), or “Sexual Well-being” (1.42: 0.78–2.58).

**Conclusion:**

The present study indicates better outcomes of HRQoL for breast cancer patients treated with OBS as compared to patients treated with BCS. There was no increase in physical discomfort among OBS patients despite more extensive surgery.

## Introduction

Breast-conserving surgery (BCS) followed by adjuvant radiotherapy, is documented to be equal to mastectomy with regard to oncological outcomes [1–3], and has to a large extent replaced total mastectomy in the last few decades. Oncoplastic breast surgery (OBS) was developed with the aim of further improving the esthetic and functional outcomes of BCS [4–7], as these affect health-related quality of life (HRQoL). However, only a few studies have addressed patient-reported outcomes (PROs), e.g., physical and psychosocial well-being, following OBS or among patients treated with conventional BCS [8, 9].

Several studies have evaluated oncoplastic surgery as a concept [4, 5, 7, 10], others have considered surgical techniques [4, 10], postoperative complications [6, 10, 11], as well as oncological [6, 11, 12] and esthetical outcomes [6, 11, 13–15]. With the development of the Breast-Q™ [16, 17] and recently the Breast-Q™ BCT module, a validated instrument for patient-reported outcome measures (PROMs) is now available.

OBS can be defined as level I and level II surgical techniques [7, 18] and OBS can be considered as an extension of conventional BCS [10]. By applying OBS in BCS, a larger number of patients may achieve good esthetic and functional outcomes, and some may also escape mastectomy [6, 7, 11, 19, 20].

The aim of this study was to investigate whether OBS improves HRQoL in patients undergoing BCS. For this purpose, we used the Breast-Q™ BCT module.

## Materials and methods

### Patients

Patients treated for invasive primary breast cancer in the period 1 January 2008 to 31 December 2013 were recruited from two cohorts. Patients in the OBS cohort (*n* = 236) were mainly recruited from the Southern Region of Denmark, while patients in the control cohort, the BCS cohort (*n* = 1423), were recruited from the Northern Region of Denmark. Clinical data were collected from the Danish Breast Cancer Cooperative Group registry (DBCG registry) [21] and the Danish Cause of Death Registry (DAR) [22]. In 2016, patients were sent a survey including the Breast-Q™ BCT postoperative module and a study-specific questionnaire (SSQ).

### Oncoplastic breast surgery cohort

**Fig. 1:**
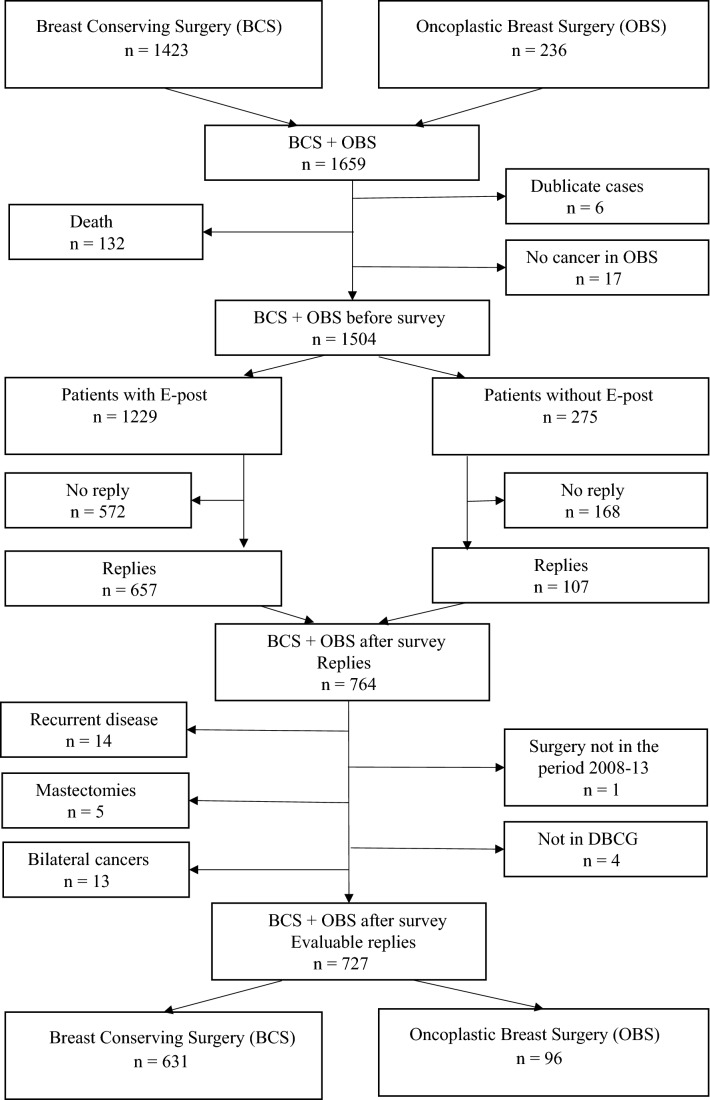
Breast-Conserving Surgery (BCS) and Oncoplastic Breast Surgery (OBS) cohorts

Patients treated with OBS were consecutively registered in the research database at the Hospital of Southwest Jutland, which is approved by the Danish Data Protection Agency [23], and 236 patients were registered (Fig. 1). Of these, 17 patients were not registered in the research database with primary invasive breast cancer and 6 cases were double entries to the database. According to data from the Danish Cause of Death Registry (DAR) [22] 13 patients had died at the time of the survey. In all, 200 patients remained in the OBS cohort at the time of the survey.

Patients in the OBS cohort were treated with either level I or II oncoplastic surgery [7, 18]. In this study, we define level I OBS as the adaptation of glandular tissue with minor mobilization of glandular tissue with or without repositioning of the NAC, and Level II OBS as reduction (therapeutic) mammoplasty, volume displacement and volume replacement techniques. The latter include reconstructions with perforator-based flaps [24, 25] and muscle sparing latissimus dorsi flaps [26]. Furthermore, the majority of the patients treated with level II surgery, i.e., patients who had a therapeutic mammoplasty or reconstruction using displacement techniques, had simultaneous contralateral surgery to achieve symmetry. Patients operated with mastectomy followed by an immediate autologous or implant reconstruction were not included in this study.

### Breast-conserving surgery cohort

All patients diagnosed with primary invasive breast cancer treated with BCS in the Region of Northern Denmark from 1 January 2008 to 31 December 2013 were identified as a consecutive population-based cohort in the national DBCG registry [21] (*n* = 1423) (Fig. 1). Data from the Danish Cause of Death Registry (DAR) [22] showed that 119 patients had died at the time of the survey, and in all, 1304 patients remained in the BCS cohort. OBS was not implemented in the Northern Region of Denmark as a routine surgical procedure during the study period.

### Patient survey

The survey included the Breast-Q™ BCT postoperative module and a study-specific questionnaire (SSQ). The SSQ included the completion of an informed consent and selected patient characteristics. Patients included in the remaining OBS and the BCS cohorts were sent the survey (*n* = 1504). A digital letter was posted to the patients in March 2016 from the Hospital of Southwest Jutland by E-post with information on the research project and a digital link to the survey (*n* = 1229). E-post is a Danish web-application developed for secure digital communication between citizens, companies and public authorities i.e., the health care system. E-post is compulsory in Denmark for all citizens, although some citizens are allowed to have only a postal address, e.g., due to lack of computer skills. A reminder was sent after four weeks to patients who had not answered. Patients who were not available by E-post were simultaneously mailed a letter with the same survey in paper form to their postal address (*n* = 275). No reminder was sent in their cases. Data were collected using the Research Electronic Data Capture (REDCap) [27] database, designed for this study. Access to REDCap [27] was licensed by the Odense Patient data Explorative Network (OPEN) University of Southern Denmark [28]. Surveys returned in paper form were transferred to the REDCap [27] database.

### Study population

In all, 764 patients replied the survey. Based on a second DBCG registry dataset form, 2017 patients were excluded if they at the time of the survey had had recurrence of the disease, a secondary mastectomy were registered with bilateral cancer, did not have surgery in the period 2008 to 2013 or if the patients were not registered in the DBCG registry (Fig. 1).

In the BCS and the OBS cohorts there were 631 and 96 evaluable replies, respectively. The total response rate for evaluable replies was 48.3% (727/1504), while the response rates for the BCS and OBS cohorts were 48.4% (631/1304) and 48.0% (96/200), respectively.

Patients in the BCS cohort underwent surgery in the Northern region of Denmark. Patients in the OBS cohort underwent surgery in the Region of Southern Denmark at the Hospital of Southwest Jutland, Esbjerg, (1 January 2008–31 December 2013; *n* = 40), at the Hospital of South Jutland, Aabenraa (1 October 2010–31 December 2013; *n* = 48), or at Privathospitalet Hamlet, Copenhagen (1 January 2008–31 December 2010; *n* = 8). Among patients in the OBS cohort, 32 had level I surgery. Sixty-four patients had level II surgery and 32 of them had contralateral surgery for symmetry. The mean follow-up time among all 727 patients was 60.8 months (range 26–100).

### Danish breast cancer cooperative group registry

The national clinical database**,** the DBCG registry [21], includes data from all departments of radiology, breast surgery, pathology and oncology in Denmark involved in the diagnosis and treatment of breast cancer. Data include information on patient age, type of breast cancer diagnosis, time of diagnosis, tumor characteristics, surgical treatment, oncological adjuvant therapy and information on recurrent disease and death. The use of oncoplastic surgical techniques has been registered since 1 July 2010. With the permission of the Danish Clinical Registries [29], the Danish National Board of Health [29], data for patients in the OBS cohort and the BCS cohort, were identified in the DBCG registry [21]. The present study used data for the patients until 3 January 2017. We used one data set retrieved in 2014 and one retrieved in 2017. Data from 2014 were used to identify patients in the BCS cohort in the DBCG registry [21] with primary breast cancer. Data from 2017 were used to exclude replies from patients who at the time of the survey had had a recurrent disease, bilateral cancer, a mastectomy, did not have surgery in the period 2008 to 2013 or were not registered in the DBCG registry. Data on patient, tumor, and treatment characteristics were also retrieved from the DBCG registry from 2017.

### Patient-reported Outcome Measure (PROM)—the Breast-Q™

The Breast-Q™ is a disease-specific validated questionnaire for evaluating PRO [16]. The linguistically validated Danish version of the Breast-Q™ BCT postoperative module includes 10 domains. In the present study, we selected the domains with regard to the patient-reported outcome of treatment, namely “Satisfaction with breast”, “Psychosocial Well-being”, “Sexual Well-being” and “Physical Well-being”. Permission to use the Danish version of the questionnaire was granted by the MAPI Research Trust Institute [30] which administers the Breast-Q™. Breast-Q™ data are transformed into scores ranging from 0 to 100 according to the guidelines for the Breast-Q postoperative module. Higher scores represent more favorable outcomes.

### Characteristics of patients, tumors, and treatments

Several factors may affect the patient-reported outcome. In the present analysis, we used the a priori selected patient characteristics: age at surgery, follow-up time, menopausal status, chest measurement, bra cup-size, BMI, smoking habits, as well as marital status, education and living arrangement. Furthermore, the following tumor and treatment characteristics were selected: TNM-classification, lumpectomy size, tumor location, radiotherapy, chemotherapy, endocrine therapy and immunotherapy, and axillary surgery. These data were mainly collected from the DBCG registry [21] whereas complementary data were collected from the SSQ.

### Statistical methods

The OBS and BCS cohorts were compared using univariate logistic regression analyses for the scores for each domain in the Breast-Q BCT module. The analyses were conducted separately for the OBS cohort including level I and II oncoplastic procedures and for the OBS cohort only including level II procedures. The scores in the Breast-Q domains (0–100) for the BCS cohort were transformed from a linear variable into a binary variable by the median score used for the cut-off value for each domain as the dependent variable, i.e., scores lower than the median score were considered less favorable and higher scores as more favorable. The risk of a better outcome was compared between the OBS and BCS cohorts as the logistic regression analysis yielded odds ratios (OR) with 95% confidence intervals. Factors that might affect the patient-reported outcome, i.e., patient, tumor and treatment characteristics, were selected à priori and included in a second multivariate logistic regression model. We performed sensitivity analyses excluding patients registered in the DBCG registry as treated with OBS from 1 July 2010 to 31 December 2013 in the BCS cohort (*n* = 24). We also compared responders (*n* = 727) to non-responders (*n* = 683) in order to check for potential selection bias. This analysis included age at surgery and the same tumor characteristics and treatment factors as used in the analyses of responding patients. Data were analyzed using IBM–SPSS Statistics version 24.0, IBM Corp., US.

### Ethics

The study was approved by The Regional Committee on Health Research Ethics for Southern Denmark. The study was submitted for evaluation to The Regional Ethical Review Board in Lund, Sweden, as the research was conducted at Lund University, Sweden, but their approval was not required (Dnr.2014/882). The research database identifying OBS patients was approved by the Danish Data Protection Agency [23].

## Results

### Patient, tumor, and treatment factors

Compared with the BCS cohort, patients in the OBS cohort were younger, more often never-smokers or non-smokers at the time of surgery, and had a lower BMI (Table 1). Notably, they had larger tumors, larger lumpectomies, were more often treated with chemotherapy, endocrine therapy and axillary clearance, and nearly all had been treated with radiotherapy (Table 2). This indicates that the patients in the OBS cohort had more advanced cancers than those in the BCS cohort.

**Table 1:** Patient characteristics

Covariates	Category	BCS	OBS	Total
		*n* = 631	*n* = 96	*n* = 727
		Column percent*		
Age at surgery (year)	< 50	15.5	25.0	16.8
	≥ 50 to < 65	56.6	52.1	56.0
	≥ 65	27.9	22.9	27.2
Menopause	Premenopausal	23.6	34.4	25.0
	Postmenopausal	73.9	63.5	72.5
Bra size	A	8.7	13.5	9.4
	B	31.2	26.0	30.5
	C	26.1	24.0	25.9
	D	18.5	16.7	18.3
	E	4.1	6.3	4.4
	≥ F	5.1	8.3	5.5
	Missing	6.2	5.2	6.1
Chest measurement (cm)	≤ 82	20.9	21.9	21.0
	83–87	20.0	25.0	20.6
	88–92	13.8	15.6	14.0
	93–97	8.4	7.3	8.3
	≥ 98	15.1	10.4	14.4
	Missing	21.9	19.8	21.6
BMI	< 25	36.8	41.7	37.4
	25.0–29.9	35.3	39.6	35.9
	≥ 30	17.6	16.7	17.5
	Missing	10.3	2.1	9.2
Ever smoker	Non-smoker	40.6	57.3	42.8
	Smoker	53.3	42.7	53.6
Smoking at surgery	Non-smoker	77.7	86.5	78.8
	Smoker	19.5	13.5	18.7
Marital status	Single	7.9	11.5	8.4
	Married	68.3	69.8	68.5
	Separated	0.6	1.0	0.7
	Divorced	9.2	10.4	9.4
	Widow	13.3	7.3	12.5
Living arrangement	Living alone	23.6	18.8	23.0
	Cohabiting	69.6	77.1	70.6
	Others	3.2	1.0	2.9
Educational level (year)	Primary school	20.8	12.5	20.6
	Secondary school	10.0	12.5	10.3
	Short (2)	32.3	22.9	31.1
	Medium (3–4)	31.9	37.5	32.6
	Higher (3–6)	4.3	7.3	4.7

*Column percent does not always add up to 100% as missing data are only shown if > 5%

**Table 2:** Tumor characteristics and treatment factors

Covariates	Category	BCS	OBS	Total
		*n* = 631	*n* = 96	*n* = 727
		Column percent*		
Tumor size	T1 ≤ 20 mm	84.0	62.5	81.2
	T2 21–50 mm	15.7	34.4	18.2
	T3 ≥ 50 mm	0.3	0.0	0.3
Lumpectomy size (cm^3^)	< 50	20.0	15.6	19.4
	50–99	30.7	26.0	30.1
	100–199	33.0	32.3	32.9
	≥ 200	14.9	21.9	15.8
Tumor location	Upper lateral	37.9	40.6	38.2
	Upper medial	14.6	8.3	13.8
	Lower lateral	8.9	9.4	8.9
	Lower medial	7.0	6.3	6.9
	Central	6.2	15.6	7.4
	Overlapping regions	23.8	19.8	23.2
Axillary dissection	No	66.2	52.1	64.4
	Yes	33.8	44.8	35.2
Radiotherapy	No	4.4	2.1	4.1
	Yes	95.6	97.9	95.9
Chemotherapy	No	58.6	49.0	57.4
	Yes	41.4	51.0	42.6
Endocrine therapy	No	42.3	34.4	41.3
	Yes	57.7	65.6	58.7
Immune therapy	No	91.1	89.6	90.9
	Yes	8.9	10.4	9.1

*Column percent does not always add up to 100 as missing data are not shown

### Psychosocial well-being

The median score for the domain “Psychosocial Well-being” was 82 with a response rate of 99.3%. There was a better outcome for the OBS cohort (OR 2.15: 1.25–3.69) (Table 3). When comparing the OBS cohort including only level II oncoplastic surgery with the BCS cohort, we found a difference that was even stronger (OR 2.67: 1.37–5.20).

**Table 3:** Odds ratios for and adjusted odds ratios for scores in Breast-Q modules “Psychosocial Well-being”, “Physical Well-being”, “Sexual Well-being”, and “Satisfaction with Breasts” for BSC and OBS level I + II and BCS and OBS level II

Domain	Cohort	All	Below median	Above median	OR	OR^a^
Psychosocial Well-being	BCS	627	311	316	1.00	1.00
Median = 82	OBS level I + II	95	38	57	1.48 (0.95–2.29)	2.15 (1.25–3.69)
	BCS	627	311	316	1.00	1.00
	OBS level II	63	23	40	1.71 (1.00–2.96)	2.67 (1.37–5.20)
Physical Well-being	BCS	623	277	346	1.00	1.00
Median = 78	OBS level I + II	95	50	45	0.72 (0.47–1.11)	0.83 (0.50–1.39)
	BCS	623	277	346	1.00	1.00
	OBS level II	63	32	31	0.78 (0.46–1.30)	0.94 (0.50–1.74)
Satisfaction with Breast	BCS	626	308	318	1.00	1.00
Median = 74	OBS level I + II	95	48	47	0.94 (0.61–1.45)	0.95 (0.57–1.59)
	BCS	626	308	318	1.00	1.00
	OBS level II	63	29	34	1.13 (0.67–1.90)	1.25 (0.67–2.33)
Sexual Well-being	BCS	431	205	226	1.00	1.00
Median = 58	OBS level I + II	75	33	42	1.15 (0.71–1.89)	1.42 (0.78–2.58)
	BCS	431	205	226	1.00	1.00
	OBS level II	50	21	29	1.25 (0.69–2.27)	1.86 (0.90–3.83)

^a^Adjusted for age, follow-up time, menopausal status, T-classification, lumpectomy size, tumor location, bra size, chest measurement, BMI, radiotherapy, chemotherapy, endocrine therapy, immunotherapy, axillary clearance, smoking, marital status, living arrangement and education

### Physical well-being

The median score for “Physical Well-being” was 78 with a response rate of 98.7%. There was no statistically significant difference between OBS and BCS patients as the OR was 0.83 (0.50–1.39) (Table 3). When only level II surgery was included in OBS, the OR was even closer to 1.00 (0.94: 0.50–1.74).

### Satisfaction with breast

The median score for the domain “Satisfaction with Breast” was 74 with a response rate of 99.2%. The adjusted OR showed no statistically significant difference between the OBS cohort and the BSC cohort (Table 3). This was also true when only level II OBS patients were considered.

### Sexual Well-being

The median score for the domain “Sexual Well-being” was 58 with a response rate of only 69.6%. There was no statistically significant difference between OBS and BCS (Table 3). Including only level II surgery indicated a slightly better outcome in the OBS group, although not statistically significant (OR 1.86: 0.90–3.83).

### Sensitivity analysis and analysis comparing responders with non-responders

Sensitivity analysis excluding patients from the BCS cohort who were registered in the DBCG registry as treated with OBS in the period 1 July 2010 to 31 December 2013 (*n* = 24), showed all the same results (data not shown). Responders and non-responders were very similar with regard to age, tumor characteristics and treatment factors (Table 4).

**Table 4:** Patient and tumor characteristics and treatment factors for the responder and non-responder cohorts

Covariates	Category	OBS		BCS	
		Responders	Non-responders	Responders	Non-responders
		*n* = 96	*n* = 71	*n* = 631	*n* = 612
		Column percent*			
Age at surgery (years)	< 50	25.0	18.3	15.5	12.7
	≥ 50 to < 65	52.1	52.1	56.6	50.0
	≥ 65	22.9	29.6	27.9	37.3
Tumor size	T1 ≤ 20 mm	62.5	63.4	84.0	82.2
	T2 21 – 50 mm	34.4	33.8	15.7	17.3
	T3 ≥ 50 mm	0.0	0.0	0.3	0.3
Lumpectomy size (cm^3^)	< 50	15.6	12.7	20.0	20.9
	50–99	26.0	32.4	30.7	27.8
	100–199	32.3	26.8	33.0	30.9
	≥ 200	21.9	25.4	14.9	17.5
Tumor location	Upper lateral	40.6	32.4	37.9	41.0
	Upper medial	8.3	12.7	14.6	11.4
	Lower lateral	9.4	11.3	8.9	10.8
	Lower medial	6.3	12.7	7.0	6.2
	Central	15.6	7.0	6.2	5.4
	> 1 region	19.8	22.5	23.8	22.1
Axillary dissection	No	52.1	59.2	66.2	64.1
	Yes	44.8	39.4	33.8	35.8
Radiotherapy	No	2.1	5.6	4.4	4.2
	Yes	97.9	94.4	95.6	95.8
Chemotherapy	No	49.0	56.3	58.6	69.4
	Yes	51.0	43.1	41.4	30.6
Endocrine therapy	No	34.4	35.2	42.3	40.8
	Yes	65.6	64.8	57.7	59.2
Immune therapy	No	89.6	90.1	91.1	94.3
	Yes	10.4	9.9	8.9	5.7

*Column percent does not always add up to 100 as missing data are not shown. Patients in the Responder cohort (*n* = 764) and Non-responder cohort (*n* = 1504–764 = 740) were excluded (Responder cohort (*n* = 37) and Non-responder cohort (*n* = 57)) if the patients were not registered in the DBCG registry, did not have surgery in the study period, did not have breast cancer, had bilateral cancers or a secondary breast cancer (bilateral event) or if they had a recurrent disease or had undergone a mastectomy before the survey (Fig. 1)

## Discussion

In this study, we evaluated the outcome of OBS compared with BCS using the Breast-Q™ BCT postoperative module. We found that patients treated with OBS had a better “Psychosocial Well-being “. However, no significant differences were found for the domains “Physical Well-being”, “Satisfaction with Breast” or “Sexual Well-being”.

Evaluation of surgical treatment of breast cancer by oncological outcomes remains essential. However, the quality of the health care services provided also needs attention. There is now an increasing demand to evaluate how patients perceive the results of treatment, i.e., PRO [8, 9, 19]. In this study we took advantage of Breast-Q™ which can be used as a standardized and validated instrument for evaluation of HRQoL in patients operated for breast cancer [16, 31–33].

Although the esthetic and functional outcome of OBS compared with BCT has been investigated before [15], there have been few studies comparing HRQoL between OBS and BCS so far. The Breast-Q™ BCT pre- and postoperative module was introduced in 2015. One year later, O´Connell et al. [8] published their initial experience with the full BCT postoperative module including 200 patients, thus establishing a benchmark for future research. However, few studies have addressed the HRQoL outcome of BCS [8, 9, 16] and OBS [34] or both. Compared with previous studies, patients included in the OBS cohort in this study represent the full spectrum of OBS, i.e., level I and II surgery, and the sample is therefore not restricted to one surgical procedure such as the therapeutic mammoplasty technique [34].

In the domain “Psychosocial Well-being” we found a median score of 82, similar to the results of O´Connell [8] and Dahlbäck [35], using Breast-Q™ for evaluation of the outcome of BCT, while Langendiik [36] found a mean score at 70.1 and Vesprini [9] found a mean score at 73.5. In our study we found a statistically significant better outcome for the OBS cohort, including level I and II surgery, compared with the BCS cohort. The difference was strengthened by including only level II surgery from the OBS cohort.

In their analyses of the domain “Physical Well-being” after BCT with Breast-Q™, Langendjik [36] and Vesprini [9] found mean scores of 71.2 and 74, respectively. A slightly higher score of 75 was reported by O`Connell [8]. The median score of 78 in this study reflects a low grade of physical discomfort and there was no statistically significant difference between OBS and BCT. One could have expected a lower score for the OBS cohort, particularly in cases of level II surgery as this surgery is more extensive and often involves the contralateral breast. On the contrary, there was a slight difference in the figures, indicating better outcomes for OBS compared with BCS.

In the analysis of the domain “Satisfaction with Breast” Vesprini [9] and Langendjik [36] found mean scores of 59.3 and 65.7, respectively, while Dahlbäck [35] and O´Connell [8] found median scores of 66 and 68, respectively. Hence, our median score of 74 in the present study means our scores are higher than those reported previously and they indicate a higher degree of satisfaction with the breast. High scores generally imply that possible differences are difficult to detect and, consequently, we found no difference between OBS and BCS. However, when only level II surgery was considered, a tendency toward a better outcome in the OBS group was noted.

The domain “Sexual Well-being” had a markedly lower median score of 58 and, furthermore, the response rate was low at 69.6%. This pattern has also been seen in previous studies investigating BCT [8, 9, 36], and it seems to be a general issue for this domain. Therefore, results must be interpreted with caution. We found no difference between OBS and BCS. However, when only level II surgery was considered, a slight tendency toward a better outcome in the OBS group was noted.

In summary, the results show that patients treated with OBS reported statistically better psychosocial health than those treated with BCS. Patients treated with OBS also scored slightly higher for the domains “Satisfaction with breasts” and “Sexual Well-being” particularly when the analyses only included level II OBS, although the difference was not statistically significant. Notably, the results in the domain “Physical Well-being” showed no significant differences despite the fact that patients treated with OBS had more extensive and often bilateral surgery.

A methodological issue to be considered is the definition of OBS. In studies published in the last few decades, the definition of OBS has varied, making it difficult to compare the outcome results from different studies. In the present study, we have based our definition on that proposed by Clough et al. [7], which is widely accepted. In the publication by Chatterjee et al. [18], a consensus definition and classifications system, developed by the American Society of Breast Surgeons, was presented which was strongly influenced by Clough’s definition [7]_._ By using a widely accepted definition of OBS we hope that the results from the present study can be used for comparison with future studies.

Another methodological issue to be considered is the PROM instrument chosen. The Breast-Q™ BCT module is now widely accepted as a valuable PROM instrument for breast-conserving surgery [31, 32,37], which is why we have chosen the Breast-Q™ BCT postoperative module.

Furthermore, the validity of the data used must be considered. Data in the research database were only used for identification of patients treated with OBS. To avoid misclassification of patient, tumor and treatment characteristics, all data for both the OBS and the BCS cohorts were obtained from the national DBCG registry [21]. In a recent study by Cronin-Fenton et al., [38] the authors conclude that DBCG data are valid for epidemiological studies of breast cancer treatment; thus, we believe that the validity of the data is good.

To avoid confounding by indication, the patients in the OBS and BCS cohorts were recruited from different geographical regions. The unselected demographic BCS cohort thus also possibly included some more advanced cases that might have been selected for OBS. In the early study period, such cases were not identified, but from 1 July 2010, 24 cases could be identified in the DBCG registry. Sensitivity analyses excluding these patients did not alter the results. The OBS cohort may include patients with relatively more advanced disease, i.e., the OBS cohort could include patients with tumor and treatment characteristics known to predict less satisfying outcomes of HRQoL. By adjusting for these variables in the statistical analyses, we have reduced this potential selection bias.

Response rates in other surveys are reported to be between 31% [9] and 76% [35]. With a total response rate in our study of 48.3% for evaluable replies (OBS cohort 48.4%, BCS cohort 48.0%) we find our response rate acceptable. The analysis comparing patients in the responder and the non-responder cohorts showed only minor differences, that is, the responder cohort is considered representative of the survey cohort, showing no selection bias.

## Conclusion

The present study indicates better outcomes of HRQoL for breast cancer patients treated with OBS as compared to patients treated with BCS. There was no increase in physical discomfort among OBS patients despite more extensive surgery.
